# sSfS: Segmented Shape from Silhouette Reconstruction of the Human Body

**DOI:** 10.3390/s22030925

**Published:** 2022-01-25

**Authors:** Wiktor Krajnik, Łukasz Markiewicz, Robert Sitnik

**Affiliations:** 1 Mnemosis S. A., 8 Józefa Str., 31-056 Krakow, Poland; w.krajnik@mnemosis.pl (W.K.); lukasz.markiewicz.dokt@pw.edu.pl (Ł.M.); 2 Institute of Micromechanics and Photonics, Warsaw University of Technology, 8 Sw. Andrzeja Boboli Str., 02-525 Warsaw, Poland

**Keywords:** Shape from Silhouette, visual hull, human body segmentation, 3D reconstruction, pose estimation, volumetric methods, computer vision, multi-view images

## Abstract

Three-dimensional (3D) shape estimation of the human body has a growing number of applications in medicine, anthropometry, special effects, and many other fields. Therefore, the demand for the high-quality acquisition of a complete and accurate body model is increasing. In this paper, a short survey of current state-of-the-art solutions is provided. One of the most commonly used approaches is the Shape-from-Silhouette (SfS) method. It is capable of the reconstruction of dynamic and challenging-to-capture objects. This paper proposes a novel approach that extends the conventional voxel-based SfS method with silhouette segmentation—segmented Shape from Silhouette (sSfS). It allows the 3D reconstruction of body segments separately, which provides significantly better human body shape estimation results, especially in concave areas. For validation, a dataset representing the human body in 20 complex poses was created and assessed based on the quality metrics in reference to the ground-truth photogrammetric reconstruction. It appeared that the number of invalid reconstruction voxels for the sSfS method was 1.7 times lower than for the state-of-the-art SfS approach. The root-mean-square (RMS) error of the distance to the reference surface was also 1.22 times lower.

## 1. Introduction

In past decades, the detailed high-resolution three-dimensional (3D) scanning of human body shapes has rapidly developed. Reconstruction accuracy, quality, and speed have improved significantly over recent years to the point where four-dimensional (4D, i.e., 3D over time) dynamic movement scanners (e.g., Diers International GmbH [1,2], 3dMD LLC [3,4], and Microsoft Corporation [5]) have become an industry standard. Both dynamic and static whole-body scanners have countless applications, such as in special effects in movies/video games [6], in human body pose and deformation analyses for medical diagnostics [7,8,9], and in anthropometry [10]. However, most of these systems have various limitations, i.e., insufficient precision, high cost, and noisy or incomplete data. The latter is usually caused by inevitable body self-occlusion and results in defective data that are full of holes. Therefore, it is one of the most important areas of interest in the 3D/4D research field.

This paper presents a novel approach to addressing one of those issues, namely surface reconstruction errors. A human body geometry is obtained using a variation of the Shape-from-Silhouette (SfS) voxel-based visual hull estimation method [11]. In the proposed segmented Shape-from-Silhouette (sSfS) approach, the reconstruction is performed for each body segment separately. The independent division into body segments for each image allows for individual 3D reconstruction of shapes with fewer concavities. Consequently, after combining the reconstructed 3D partial models, the representation of the human body geometry is significantly better compared to the classic SfS method results. For silhouette estimation in images, solutions based on an existing convolutional neural network (CNN) can be used. Therefore, this technique requires only an efficient method for silhouette extraction from the image and a calibrated set of cameras. A state-of-the-art survey of other well-established methods is also presented. 

## 2. Related Works

### 2.1. 3D Reconstruction Methods Used for Human Body Scanning

The acquisition of the 3D human body shape is a demanding process, requiring a high resolution and short acquisition time due to the body’s complexity and sway. A 3D whole-body scanning approach can be developed using various measurement methods [12]. The most common ones are presented in Table 1.

Of these four methods, LT is typically the slowest in terms of acquisition time due to the utilization of a laser line that is incapable of scanning the whole human body surface at once. A single static scan takes a significant amount of time (e.g., 9 s with Vitus Bodyscan [17]) and is not suitable for dynamic motion capture [18]. Despite being faster, the ToF method provides the lowest resolution of the 3D measurement. It can be increased by adding more camera views, but it is limited by the interference of light sources on the measured subject’s surface [19]. By comparison, the SL method provides fast and comprehensive 3D shape acquisition [20]. It requires structured pattern projection. One of its drawbacks is light interference from multiple projectors distributed around the measured subject. This issue can be solved with an example solution proposed in [21], in which spectral filters were used to avoid the crosstalk between neighboring cameras and projector modules. Finally, the photogrammetry method benefits from a short image acquisition time, a lack of interference issues, and a relatively simple reconstruction process, with high overall performance [22]. Systems with fixed camera positions are emerging for the measurement of bodies in motion. One of the most common photogrammetric methods is Structure from Motion (SfM), typically followed by the use of the multi-view stereo (MVS) [23] algorithm, responsible for the densification of the SfM point cloud. Multiple open-source and commercial software packages exist for photogrammetric reconstruction, such as VisualSFM [24] and Agisoft Metashape [25]. However, the photogrammetric systems must provide enough views to avoid occlusions in the measured area, as proposed in [26]. In addition, they provide poor results for low-repeating textures, shaded areas, and reflecting spots.

### 2.2. SfS Visual Hull Estimation

Recently, many authors have proposed reconstruction methods based on a visual hull [11] extraction from a set of multi-view binary silhouette images. Visual hulls can be either computed as an exact polyhedral representation or approximated as voxels.

Polyhedral-based visual hull estimation approaches [27,28] estimate visual cones for each silhouette by casting rays from the camera center to the silhouette edge. Then, the subject’s shape is formed based on the intersection of those visual cones. The result is the surface of the subject, by design, without the concave parts of the subject. This approximation can be further enhanced by eliminating rough edges of the visual hull [29,30].

By comparison, voxel-based methods [31,32] divide the 3D space into a grid of voxels. Then, the visual hull is formed based on the voxels projected onto the silhouette images based on the voting threshold (in which each successful voxel projection receives a vote). Moreover, the SfS reconstruction method [33] proposes a solution for non-binary silhouettes containing probability maps, where the formation of the visual hull is introduced as a pseudo-Boolean optimization problem [34].

Due to the ease of computing parallelization, SfS methods are suitable for working in real time [35]. Nevertheless, such implementations are limited by the number of views and the reconstruction resolution. For example, Perez et al. in [36] achieved 30 frames per second rate in a 256 × 256 × 128 voxel resolution. Thus, the resolution achieved through real-time SfS implementation is far from meeting the demands of human body reconstruction.

This approach is not only limited, similarly to other reconstruction methods, by body self-occlusions, but also by the extraction quality of the silhouettes, which makes it even harder to properly reconstruct dynamic objects such as limbs. Corraza et al. in [37] matched rigid segments of an a priori human 3D model to visual hull reconstruction in order to estimate human poses. In contrast, Kanaujia et al. in [38] utilized a previously prepared mesh. Reconstructed points from the visual hull were segmented using mesh vertices. Then, the segmented body parts were used for human joint estimations. Other works have proposed improvements in the visual hull by refining its shape using depth information from RGB (red, green, blue) stereo image matching [39,40].

### 2.3. Parametric Body Models

In general, these methods use the input 2D image data to estimate the human body model parameters, which represent human poses and body shapes. Examples of such body models are the Skinned Multi-Person Linear (SMPL) model [41] and Shape Completion and Animation of People (SCAPE) [42]. Recent works have proven that it is possible to estimate the body shape using only a single-view video, 2D positions of human joints, or multi-view silhouette images with the SCAPE [43,44,45] and SMPL [46,47] body models. The reconstruction method proposed in [48] is an example of an encoder-decoder CNN architecture that predicts the SMPL model to fit a set of silhouettes. Alternatively, Dibra et al. in [49] presents a CNN architecture that is first trained to find a human shape model from a set of 3D meshes’ body shape invariants. Then, the human pose is estimated based on a single or two-view silhouette.

### 2.4. Deep Neural Network Architectures

The latest research shows that it is possible to perform 3D human geometry reconstruction using deep learning without a parametric body model [50]. For example, Gilbert et al. [51] used an auto-encoder architecture to estimate human body shapes by improving its volumetric visual hull reconstruction. This solution is similar to super-resolution image enhancement because it augments a coarse visual hull to a high-fidelity body model. Natsume et al. [52] generated a 3D pose from a single view to produce a set of multi-view silhouette images. Then, the silhouettes were used to estimate the final 3D human body reconstruction with deep visual hull prediction, similarly to [50]. In addition, Xu et al. in [53] estimated an actor’s rig with a multi-view photogrammetric scan and a skeleton. The proposed algorithm fits the rig to the given pose with the use of silhouette images. Finally, the solution presented in [54] introduces a coarse-to-fine 3D reconstruction method for the human body. A coarse reconstruction from images was demonstrated with the 2D feature-based Pixel-aligned Implicit Function based on Multi-scale Features (MF-PIF) method, inspired by [55], and this was then refined with a voxel super-resolution network.

### 2.5. Local Shape Approximation and Meshing

The problem of the generation of watertight 3D models is typically addressed by meshing algorithms. This includes, inter alia, screened Poisson reconstruction over a sparse point cloud [56], triangular mesh hole-filling using a moving least squares approach [57], and volumetric diffusion [58]. Modeling of the unknown underlying geometry of the cavities can also be performed with algebraic surfaces [59] or the fitting of Bezier patches [60] to the neighborhood surface points.

### 2.6. Summary

Human-body-model-based methods have huge potential and provide a great solution to attaining the complete shape of the desired pose. However, the resulting models are still not fully realistic, especially in the case of unusual body positions. In contrast, meshing and local shape methods are merely mathematical approximations, unaware of the human body’s peculiarities. Furthermore, deep-learning-based methods require significant computing resources with a large amount of graphics processing unit (GPU) memory to reconstruct the models in high resolution.

## 3. Materials

**Figure 1:**
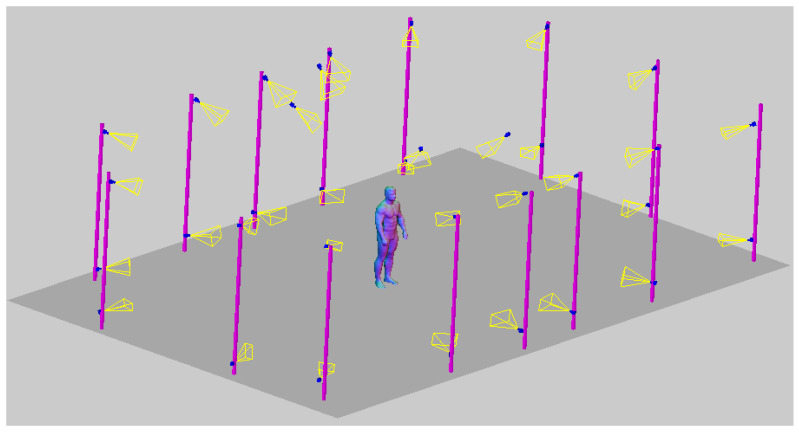
Visualized measurement scene with 34 camera distribution and sample-measured subject SfM point cloud.

For verification purposes, a dynamic fighting sequence was synchronously captured using 34 color (RGB) cameras in a 4096 × 2160 (4 K) pixel resolution. The cameras were calibrated and their positions in a global coordinate system were known. Figure 1 shows a 3D visualization of the measurement scene with a subject in the center and an even distribution of the cameras. On each of the 15 columns, 2 cameras were hung at heights ranging from 0.5 to 3 m. Additionally, 4 cameras were placed on the 3.5 m ceiling.

In order to obtain the most accurate silhouette segmentation results on the 2D images, the Omnimatte pre-trained CNN model was utilized [61]. This takes as an input a rough mask of an object, and on the output returns precisely segmented images of the object and a background. To estimate the rough mask of the object, the Omnimatte utilizes a pre-trained DeepLabV3 model [62]. The chosen CNN solution provided satisfactory results for silhouette estimation purposes. Nevertheless, it should be noted that the sSfS method is not restricted to it and, in the future, it can be replaced with a better silhouette estimation method.

As a reference, the photogrammetric reconstruction was performed using Agisoft Metashape software [25]. To ensure comparability, the same RGB images were used for both proposed and reference methods. The exemplary camera images and calculated masks from the validation dataset for 2 of 20 different subjects’ poses are shown in Table 2 with respective ground-truth 3D reconstructions. As can be clearly seen, reconstruction defects are present in the form of holes. The complete test data collection can be seen in Appendix A, in Table A1.

## 4. Methods

In this section, the details of our sSfS reconstruction method are described. First, the adaptation of the typical SfS visual hull estimation method is presented, along with the object volume estimation and voxel voting processes. Then, the sSfS method is detailed, including the novel step of human body segmentation on the silhouette images.

### 4.1. The Conventional SfS VISUAL Hull Reconstruction of the Entire Human Body

**Figure 2:**
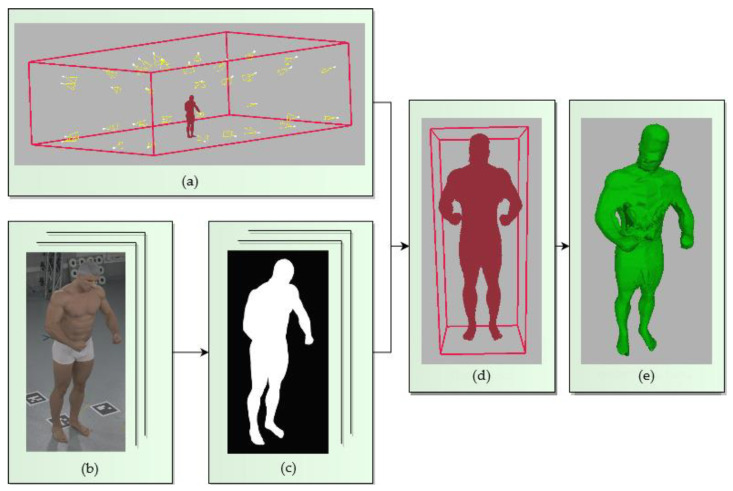
Steps involved in the conventional SfS reconstruction method of an object: (**a**) the volume of the whole reconstruction system, estimated with the cameras’ positions; (**b**) input RGB images; (**c**) silhouette images of the subject; (**d**) approximate subject volume from the transition step (see Figure 3 for details); (**e**) final visual hull estimation with the target voxel size.

The implemented SfS reconstruction approach extracts the visual hull of the measured object in the voxel representation, similarly to the state-of-the-art SfS solutions [11,36]. The visual hull is based on creating the voxel grid space in the selected volume and estimating the voxels of the reconstruction with a projection of the voxel centers on the silhouettes. The visual hull estimation steps of the SfS algorithm are shown in Figure 2.

#### 4.1.1. Calculation of the Subject’s Volume

**Figure 3:**
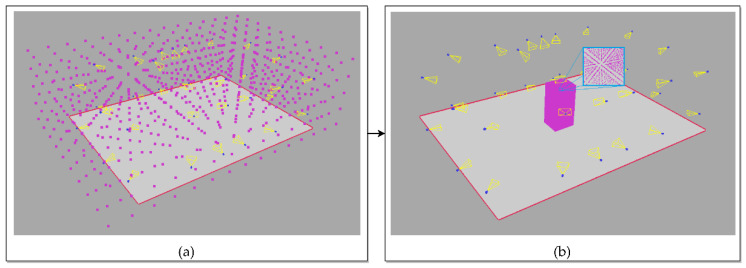
Estimation of the subject’s volume: (**a**) the initial voxel grid with voxel size D_0_ in the volume of the system cameras; (**b**) the voxel grid with voxel size D_1_ of the subject’s volume.

The output of the SfS reconstruction is a set of voxels with a known dimension D and center coordinates. Before the actual SfS reconstruction of the subject, the 3D volume of the cameras’ bounding box is divided into a voxel grid with a given single voxel size D_0_. Then, a rough reconstruction of the subject is performed inside this volume to obtain its coarse dimensions. Finally, the reconstruction of the subject is carried out with a lower voxel size D_1_ to obtain more accurate and dense results. The transition from the initial voxel size D_0_ to the target D_1_ is depicted in Figure 3. The voxel size transition allows one to solve the potential memory-overload issue, which may occur if all of the cameras’ volume was filled with a dense grid of a voxel size D_1_.

#### 4.1.2. Visual Hull Estimation with the Voxel Projections

**Figure 4:**
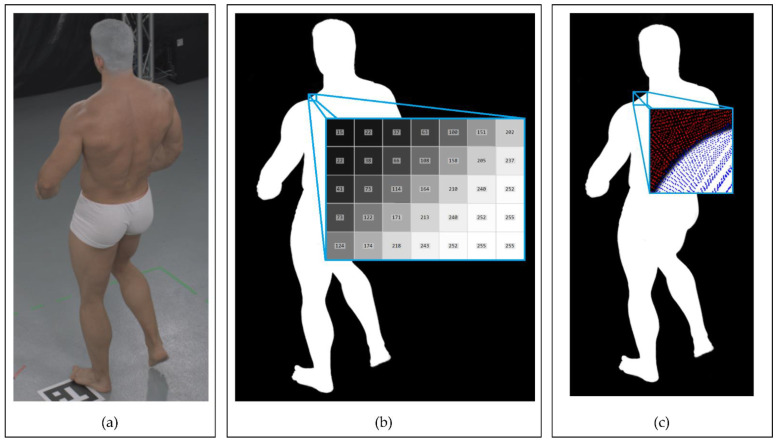
Example of Omnimatte [61] silhouette estimation performance with voxel projections onto the image (data sample #9): (**a**) input RGB image; (**b**) silhouette image with magnified silhouette quality values on its edge; (**c**) voxel center projections onto the silhouette. The projections in the image are marked with pixel-sized dots. The blue pixels represent the silhouette, and the red represent the background.

For the silhouette estimation, we decided to utilize the state-of-the-art Omnimatte CNN-based method [61], which separates objects of a specified class from the background, particularly humans. The silhouette image obtained using Omnimatte net is not binary. Instead, it contains a quality parameter that defines the probability of a correct silhouette determination in each pixel encoded as an image intensity from 0 to 255. During the projection of voxels onto the silhouettes, the algorithm counts probabilities for voxels from all of the cameras. The sum is then compared with a threshold to determine the voxels of the visual hull. Figure 4 shows the process of the silhouette estimation and the projection of the voxels onto a silhouette. The magnified areas in the projection images in Figure 4 show a rough probability transition on the edge of the silhouette.

### 4.2. sSfS Body Segment Reconstruction

The proposed coarse-to-fine sSfS method reconstructs the selected body parts separately and merges those partial results into one. Thus, each body part’s visual hull estimation algorithm is the same as for the whole-body reconstruction method shown in Figure 2. However, to do this, the 2D human body segmentation of the silhouette images is required. Therefore, a custom method was implemented to map a 3D human body segmentation to 2D silhouettes. Nevertheless, this can also be achieved with several other methods, such as CNN body segmentation based on pose estimation from images [63,64].

**Figure 5:**
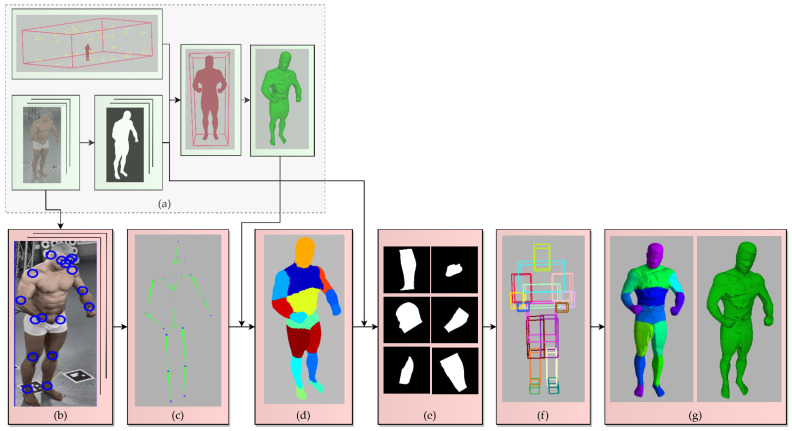
Proposed sSfS reconstruction approach flowchart. The steps added to the conventional SfS approach are highlighted in pink: (**a**) conventional visual hull estimation with silhouette images of an entire subject’s body (see Figure 3 for details); (**b**) results of the estimation of human joint positions on the 2D color images with a CNN-based Human Pose pre-trained model [65]; (**c**) retrieval of the 3D joint positions by casting the rays leading from each camera center’s 3D position to each joint and calculating the best intersections; (**d**) segmentation of the subject’s coarse 3D visual hull voxel reconstruction; (**e**) silhouette image segmentation by projecting the segment points onto the silhouettes (see Figure 6 for details); (**f**) estimation of the 3D volume of each body segment; (**g**) SfS reconstruction results for each body part separately and merged as a whole sSfS body model.

**Figure 6:**
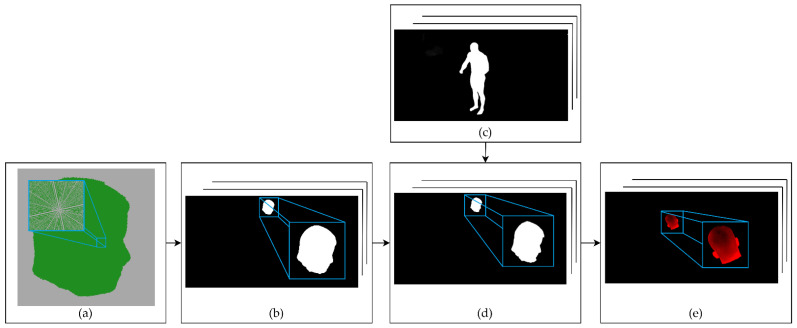
The estimation of the body segment silhouettes and their volumetric reconstructions. (**a**) Segment from the initial coarse volumetric reconstruction step with a 4 mm voxel size. (**b**) Projection of the voxels from the segment reconstruction onto the camera images for all the system’s cameras. The pixels where the projection was performed are changed to a value of 255. (**c**) Input silhouettes of the subject’s entire body from a set used to estimate an initial visual hull reconstruction. (**d**) Body segment detection for all silhouette images. The projection from step (**b**) is used as a mask for the segment on the silhouette image. (**e**) Detection of erroneous silhouettes by calculating the ratio of the segment projection pixels to the uncertain silhouette pixels, with values in the range <1, 254>.

To segment the body parts on silhouette images, the estimated positions of human joints obtained from the Human Pose CNN [65] on RGB images of the subject are used. Then, each of the approximate joint positions is found by means of ray casting from each corresponding 2D joint position to estimate the 3D joint position as the closest point to all 3D ray lines. As a result, the positions of the joints in a 3D space of cameras in the system can be estimated, as shown in Figure 5c. Next, the 3D joints are used to segment the visual hull reconstruction by assigning each voxel to the closest bone (defined as a section between two joints). However, this step could also be performed with other human body 3D segmentation approaches [66,67]. The result of the proposed 3D segmentation is shown in Figure 5d. Finally, visual hull voxels from each segment are projected onto the silhouette images to obtain segmented silhouettes, as can be seen in Figure 5e. The detailed silhouette estimation process for the segments is shown in Figure 6, with an example of head segment detection.

After the silhouette body part segmentation, each segment is reconstructed according to the conventional SfS algorithm, using the same voxel vote threshold value for the reconstruction of each body segment. Finally, the individual voxel models of body parts are merged and form the final sSfS reconstruction (Figure 5g).

As a result of the silhouette segmentation, it is possible to perform the reconstruction for each recognized body segment separately. This method allows us to find the errors in the silhouette estimation and skip the views for poorly detected body segments. To identify the erroneous segments, the ratio of the silhouette’s uncertain pixels number (pixels with intensity values in the range <1, 254>) to the segment projection pixels number (pixels with intensity value 255) is calculated. The projection image refers to Figure 6b, and the silhouette segment image refers to Figure 6d. The uncertain silhouette pixels ratio calculation is described with the following equation:

(1) $$F=\frac{M}{N}$$

where:

*F* uncertain pixels ratio,
*M* number of pixels in the silhouette segment image in range <1, 254>,
*N* number of pixels in the segment projection image with intensity 255.

By applying a simple F_t_ threshold value for each silhouette segment image, only the high-quality segments of the body silhouette are obtained for reconstruction, thus increasing the final visual hull’s quality.

### 4.3. Hardware and Software Environment

The SfS reconstruction algorithms [11] were implemented in C++, and the interface for the CNN silhouette [61] and 2D joint position estimations [65] was implemented in Python. For the method’s evaluation, we used a computer with a 2× Intel(R) Xeon(R) Silver 4215 processor @ 2.5GHz, 2× NVIDIA RTX 2080Ti and 512GB RAM.

## 5. Results

This section presents the results of the SfS and sSfS reconstructions for selected data samples compared with the SfM reference. Additionally, the difference between the SfS and sSfS outputs is presented using quality metrics. Both SfS and sSfS reconstructions were calculated with voxel sizes D_0_ 64 mm and D_1_ 4 mm for the dataset of 20 samples described in Section 3. The conventional SfS was reconstructed according to the algorithm steps in Figure 2. Then, this coarse SfS reconstruction was used in the sSfS phase, as shown in Figure 5.

### 5.1. Surface Shape Comparison

**Figure 7:**
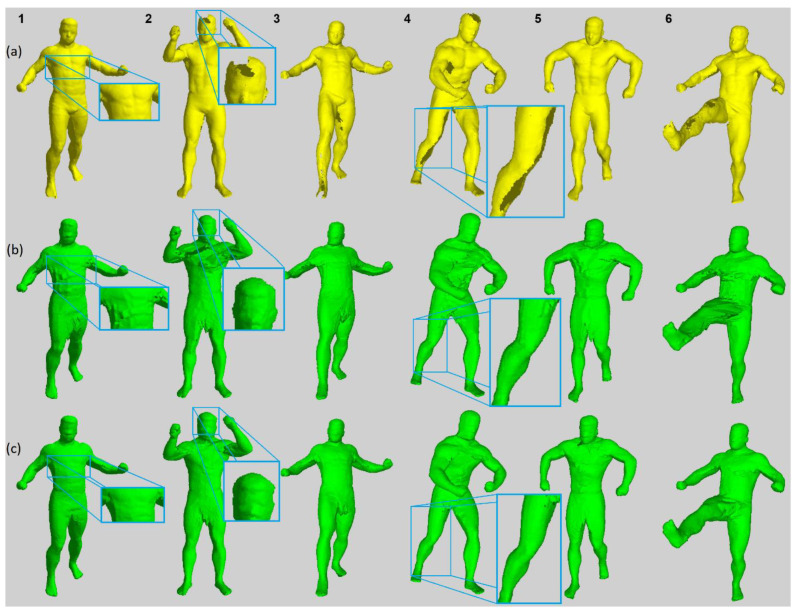
Surface reconstruction results comparison for selected dataset samples; every column ID number corresponds to a data sample from Table A1: (**a**) SfM; (**b**) SfS; (**c**) sSfS.

**Figure A1:**
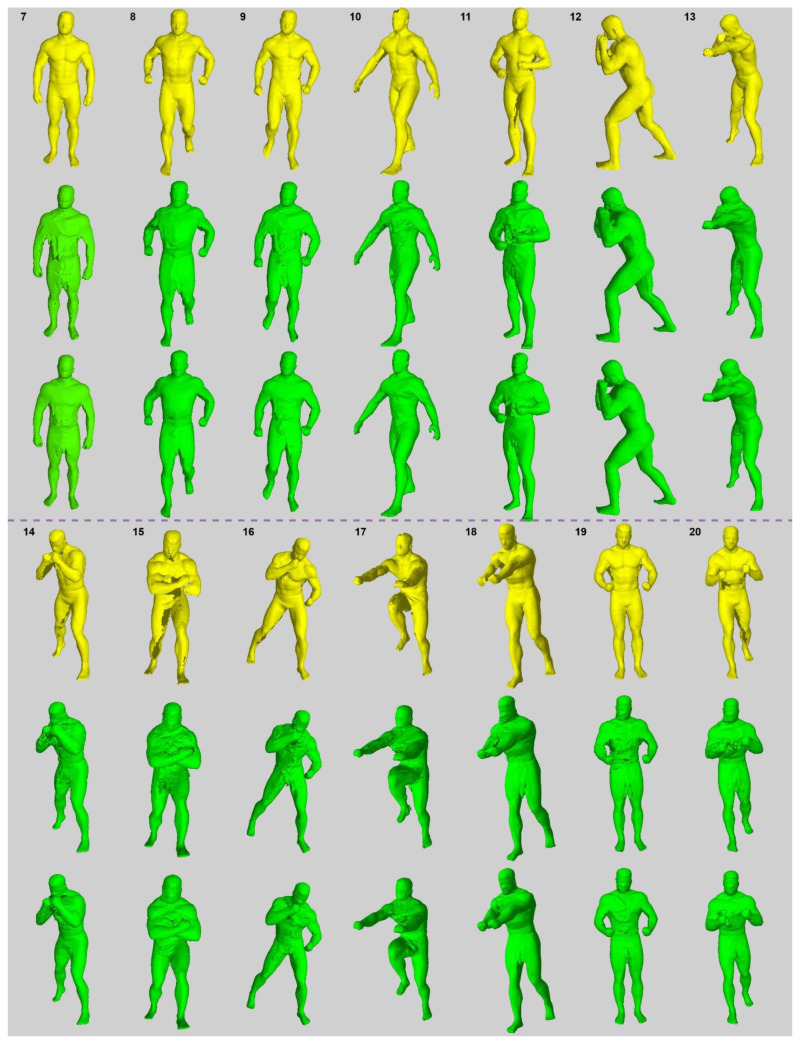
Visualization of the SfS/sSfS reconstructions for data samples #7–#20.

A visual comparison between the SfS and sSfS reconstruction surfaces is shown in Figure 7. The surface voxel of the visual hull was found using morphological erosion [68] (every eroded voxel was considered a surface voxel). The results of both methods were compared to the ground-truth SfM point cloud to assess their accuracy and their ability to fill the holes. In contrast to the SfM reference, SfS and sSfS provided results without significant distortions on the head and legs. For example, in data samples #2 and #4 in Figure 7, the SfS and sSfS correctly reconstructed the top head area, whereas the ground-truth SfM failed to do so. Similarly, the missing surface on the legs in the SfM reconstruction can be recovered in SfS and sSfS, as in data samples #3–#6 in Figure 7. In addition, the sSfS reconstruction surface was less noisy and rough than that of SfS, for example, on the chest in data sample #1 in Figure 7. The abovementioned reconstruction results for all 20 of the test poses are attached in Appendix A, Figure A1.

### 5.2. Metric Description and Results

Further quantitative analysis of the surfaces from the novel SfS and conventional sSfS was carried out in reference to the photogrammetric point clouds using two metrics:

- The number of erroneous visual hull voxels outside of the reference surface—For each voxel center, the closest point of the SfM reconstruction was found. Then, the dot product A between the vector from the voxel center to the SfM point and the SfM point’s normal vector was estimated. The voxel was considered erroneous when the value of the dot product A was less than 0. Additionally, when the distance between the voxel center and SfM point was less than the voxel size (4 mm), the voxel was not counted as erroneous.
- The distance between the SfS/sSfS reconstruction voxel and the reference cloud’s surface (point to surface, P2S)—The Euclidean 3D distance between the voxel center on the surface of the SfS/sSfS reconstruction and the closest SfM point.

#### 5.2.1. The Number of Erroneous Voxels

**Figure 8:**
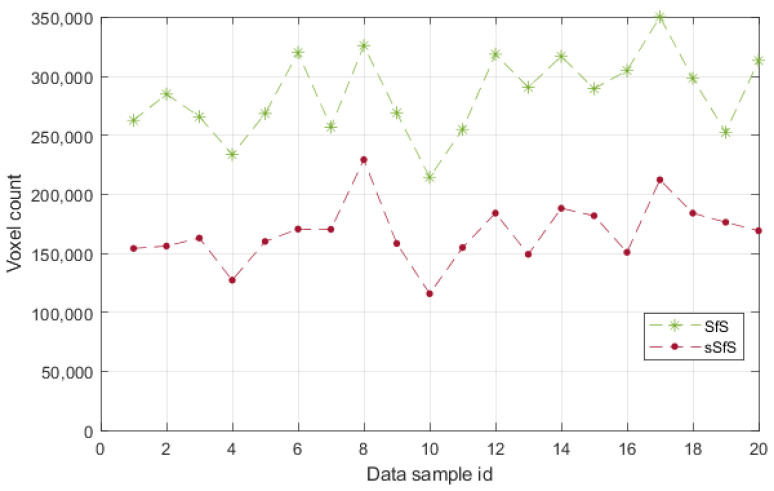
The erroneous voxel counts for SfS and sSfS reconstructions of the validation dataset.

The number of erroneous voxels provides information about the visual hull performance on the concave parts of the subject’s pose. The subject’s placement in the scene has a high impact on the number of voxels outside the reference surface, which determines the system cameras’ capability to extract the visual hull. However, this metric can show the robustness of the visual hull estimation in relation to the body part occlusions. The erroneous voxel metric results for the SfS and sSfS reconstructions for the entire dataset are presented in Figure 8. It can be seen that, in some cases, our novel sSfS reconstruction contained about two times fewer erroneous voxels than the conventional SfS results. The approximate mean number of invalid voxels for the entire validation dataset in the SfS method was 285,000, with a standard deviation of 35,000 voxels. By comparison, the average number of invalid voxels in sSfS was 168,000, with a standard deviation of 25,800 voxels. The division of those mean values shows that the sSfS allowed us to obtain 1.7-fold better results for the erroneous voxels metric.

#### 5.2.2. Surface Voxels’ Distance to the Reference

**Figure 9:**
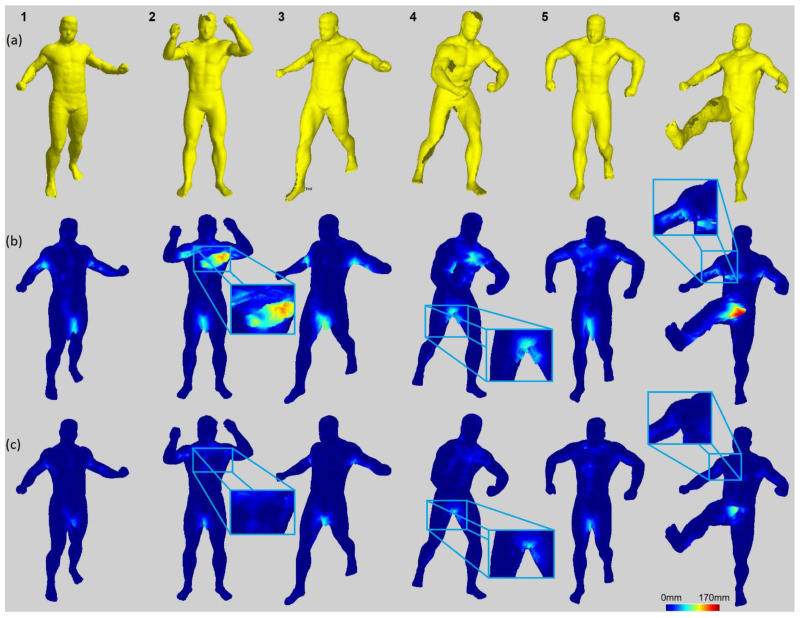
Visualization of P2S distances for SfS and sSfS results: (**a**) SfM; (**b**) SfS with P2S error map; (**c**) sSfS with P2S error map. Each data sample in the column corresponds to a data sample from Table A1; the numbers at the top of each column relate to the data sample ID.

**Figure A2:**
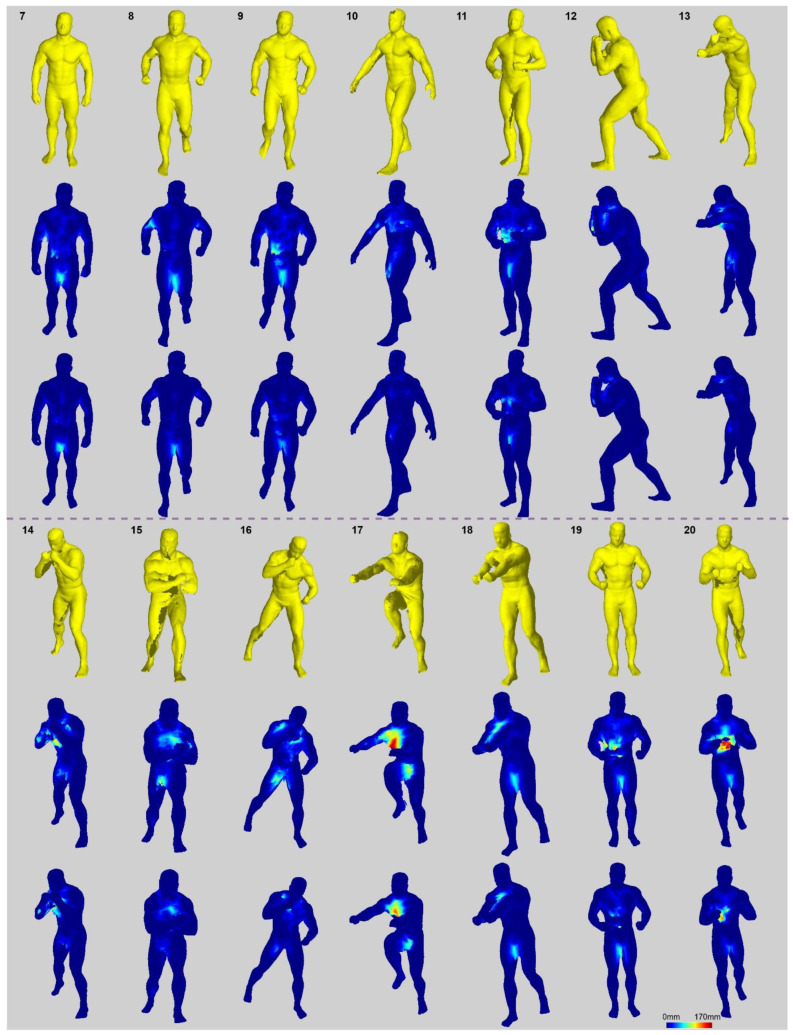
Visualization of the P2S distances for SfS/sSfS reconstructions for data samples #7–#20.

Calculating the distances of the surface voxels in relation to the reference surface allows the visualization of the visual hull error and tracking of the most problematic human body areas to reconstruct. Figure 9 shows the examples of the distance-to-surface error for both the SfS and sSfS reconstructions (the rest of the data samples’ results are shown in Appendix A, in Figure A2). In the visualization, it can be observed that the most problematic pose scenarios were observed mostly for occluded poses, especially when the limbs were near the torso area, in which both tested reconstruction approaches showed errors. Even though the results were imperfect for both approaches, the advantage of the sSfS approach is still clearly visible. Furthermore, the sSfS method achieved better results over the entire body surface. The advantage is primarily visible on the most challenging body parts, such as the armpit and crotch (magnified examples shown in Figure 9). High reconstruction quality on these body parts is crucial because the crotch and armpits are examples of the most problematic spots for SfM reconstructions.

**Figure 10:**
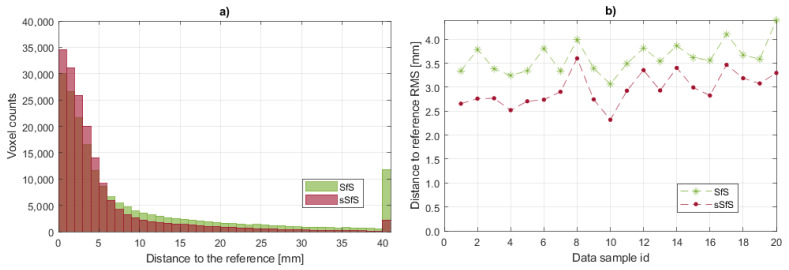
P2S error histograms: (**a**) overlapping SfS and sSfS histograms for dataset sample #6. The *x*-axis represents the histogram bin ranges with a bin of 1 mm. The last bin contains the sum of all samples with a distance >40 mm; (**b**) RMS of the P2S distances for SfS and sSfS reconstructions for the entire validation dataset.

In addition, histograms of the distances of the visual hull surface voxels to the reference surface were calculated. The example of the overlapping histograms of SfS and sSfS P2S in Figure 10a demonstrates that sSfS was characterized by more points with P2S distances up to 6 mm, in contrast to SfS, which contained more voxels with higher distance errors. Moreover, SfS had a high number of voxels with a P2S distance of more than 40 mm, which is also visible in Figure 9. Furthermore, the root-mean-square (RMS) of the P2S distances plot (Figure 10b) shows an advantage of the sSfS method on every data sample. The mean RMS of the P2S error for the SfS method was 3.62 mm, with a standard deviation of 0.34 mm for the entire validation dataset. For the sSfS method, the mean RMS was 2.92 mm, with a standard deviation of 0.32 mm. The RMS of the P2S error for the validation dataset was 1.22-fold better in the sSfS method.

### 5.3. Computation Time Comparison

The computation time of the implemented SfS reconstruction was around 30 s for a single pose, including silhouettes’ estimation. In comparison, the sSfS reconstruction took around 250 s for a single pose, involving the joint estimation and reconstruction with the coarse SfS visual hull segmentation.

## 6. Discussion

**Figure 11:**
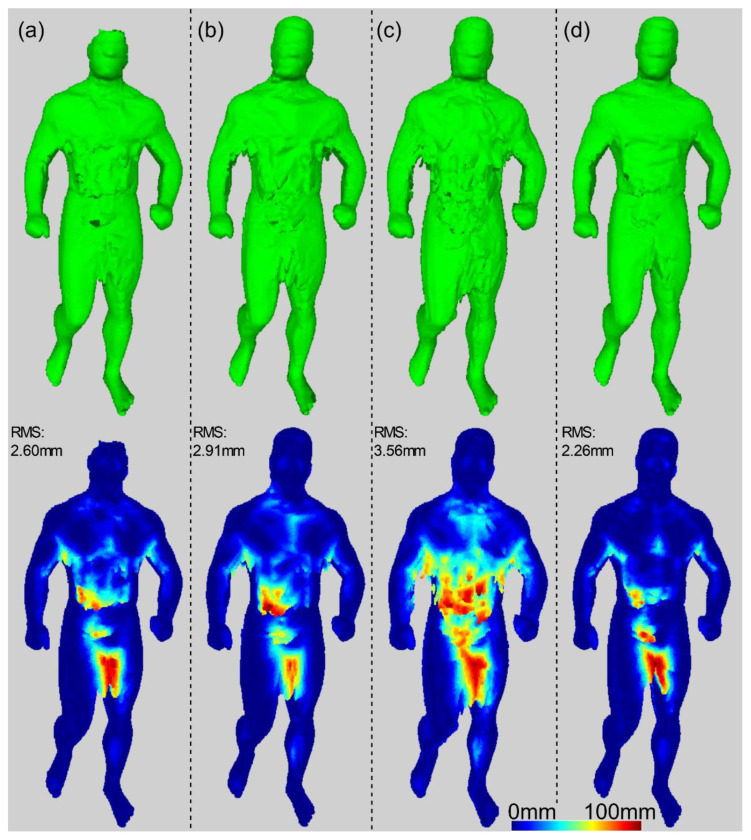
Comparison of the results of three different SfS reconstruction approaches and sSfS for data sample #9: (**a**) SfS for a high voting threshold for all views; (**b**) SfS for a high voting threshold for selected views; (**c**) SfS for a low voting threshold for all views; (**d**) sSfS.

The complete reconstruction of a subject’s body with SfS is not a trivial task, as it is prone to silhouette estimation errors. Using a simple fixed voxel vote threshold is insufficient to solve the erroneous silhouette issue. If the vote threshold is high, accurately estimated body parts are reconstructed with high quality. However, if estimation is uncertain, some body parts may not be included in the reconstruction model, as they would not accumulate enough votes. Conversely, a lower voxel vote threshold value can yield superfluous artefacts on the reconstructed surface. Another approach is to skip views containing a significant number of uncertain pixels, for example, by analyzing uncertain pixels in the silhouette. In this manner, the reconstruction quality of the body parts deteriorates, but the body shape remains undistorted. The impact of the voting threshold and the view selection on SfS in terms of reconstruction quality and surface noise is shown in Figure 11. The SfS results are also compared to those of the sSfS approach, which demonstrated the best quality regardless of the voting threshold.

**Figure 12:**
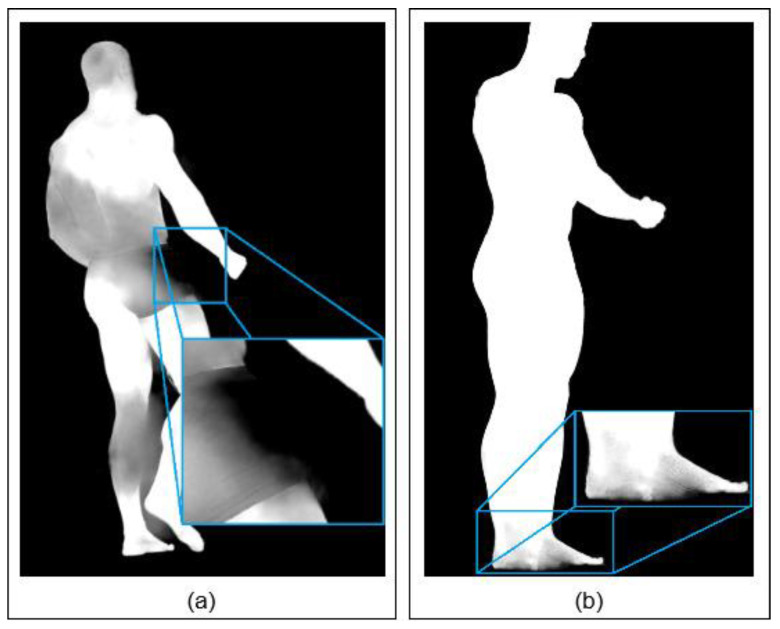
Examples of erroneous silhouette areas: (**a**) torso, left leg, and head; (**b**) feet.

Examples of silhouette estimation issues are presented in Figure 12, which shows two silhouettes containing significant distortions. The problem with uncertain silhouette estimation appears mostly in places with a low color difference between the subject’s body and the background or around the overlapping regions of body parts. However, Figure 12 shows that correctly estimated silhouette segments could be used at least partially in the sSfS reconstruction process per segment and thus increase the overall quality. For example, Figure 12a shows that the entire right arm’s silhouette is well estimated. In Figure 12b, only the silhouette part on the feet is distorted, and the rest of the silhouette can be used for sSfS reconstruction.

Despite having significantly better reconstruction quality than the conventional SfS reconstruction, the sSfS method also has some weaknesses. A primary disadvantage of the sSfS approach is its dependence on the coarse 3D body reconstruction and the subsequent segmentation. The 3D body segmentation can be erroneous in the case of noisy data or complex body poses. The sSfS method provides fewer noise voxels, but is also less universal due to the need for silhouette segmentation. By comparison, the current version of the sSfS reconstruction contains manually adjusted thresholds for voxel voting and view skipping, specifically for the example dataset. The use of manual thresholds may be an issue for larger datasets with irregular lighting or different numbers of camera views. Furthermore, the sSfS computation time can be improved by adjusting algorithms to parallel computing.

## 7. Conclusions

Detailed 3D reconstruction of the human body is a challenging task, and many possible areas exist for improvement in the existing solutions. This paper proposes a robust sSfS method that significantly enhances the conventional SfS reconstruction quality. It utilizes 2D body extraction algorithms to achieve high-quality silhouettes, in addition to human body segmentation. Performing 2D body segmentation on silhouettes enabled the introduction of the segmented Shape from Silhouette (sSfS) approach, which demonstrated robustness for silhouette estimation distortions and showed better reconstruction quality than the conventional SfS on every tested data sample. Furthermore, it was shown that sSfS can be used for the filling of cavities in the human body point cloud obtained through another reconstruction method.

Despite the promising experimental results presented in this paper, further work is required to improve the sSfS results. For example, the sSfS is dependent on the 3D segmented coarse reconstruction, which determines the quality of the segmentation of silhouettes. Therefore, the coarse SfS reconstruction method that is an input for the sSfS segmentation step can be replaced with some other reconstruction method, such as a multi-view parametric human model-based method [46] or one of the CNN-based 3D reconstruction approaches [54].

Moreover, the rapid evolution of 2D segmentation algorithms may eliminate the need for initial 3D segmentation from our pipeline. Furthermore, the development of 2D segmentation solutions may allow the sSfS to improve the reconstruction of other object classes. Another means to improve the current sSfS approach is to develop a better silhouette estimation algorithm by replacing the current method or utilizing efficient silhouette postprocessing.

## Figures and Tables

**Table 1:** A short summary of the 3D reconstruction methods used for human body scanning.

Method Name	Measurement Technique	Type of Illumination
Laser triangulation (LT) [13]	Detection of laser stripes projected onto the object’s surface	Laser
Time-of-Flight (ToF) [14]	Measurement of the depth data of the subject surface from return time of light impulse or phase shift	Infrared laser
Structured Light (SL) [15]	Analysis of a light pattern (e.g., fringes) projected onto the measured object’s surface	Structured two-dimensional (2D) pattern projected by laser or digital projectors
Photogrammetry [16]	Detection and analysis of the key point correspondences between images of the object taken from different angles simultaneously	Natural sunlight or shadowless illumination

**Table 2:** Sample test poses (2 out of the total of 20) used to validate the sSfS algorithm with corresponding RGB images, estimated silhouettes for 2 chosen cameras out of the set of 34, and photogrammetric reconstruction.

Sample RGB Images	Sample Silhouettes	Ground-Truth SfM Reconstruction
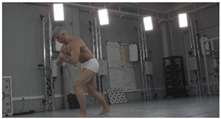	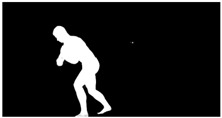	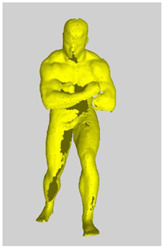
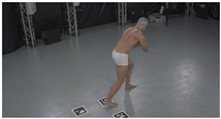	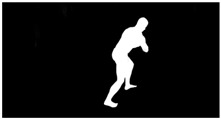	
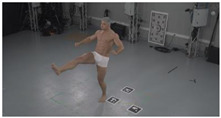	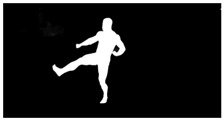	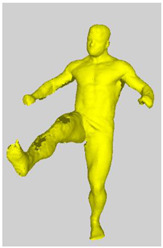
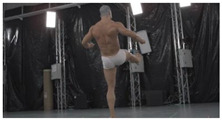	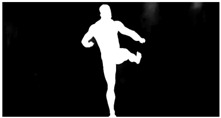	

## Appendix A

**Table A1:** Sample RGB images with estimated silhouettes for 34 cameras and SfM reconstructions used to validate the SfS/sSfS reconstruction. The dataset contains image sets and 3D scans of 20 different poses of the subject.

Id.	Sample RGB Images	Sample Silhouettes	SfM Ground-Truth Reconstruction
1			
2			
3			
4			
5			
6			
7			
8			
9			
10			
11			
12			
13			
14			
15			
16			
17			
18			
19			
20			
